# Main Colonic Metabolites from Coffee Chlorogenic Acid May Counteract Tumor Necrosis Factor-α-Induced Inflammation and Oxidative Stress in 3T3-L1 Cells

**DOI:** 10.3390/molecules29010088

**Published:** 2023-12-22

**Authors:** Luis Goya, Andrea Sánchez-Medina, Mónica Redondo-Puente, Rudolf Dupak, Laura Bravo, Beatriz Sarriá

**Affiliations:** 1 Department of Metabolism and Nutrition, Institute of Food Science, Technology and Nutrition (ICTAN-CSIC), Spanish National Research Council (CSIC), José Antonio Nováis 6, 28040 Madrid, Spain; luisgoya@ictan.csic.es (L.G.); andsan25@ucm.es (A.S.-M.); monicaredondopuente@gmail.com (M.R.-P.); lbravo@ictan.csic.es (L.B.); 2 Department of Nutrition and Food Science, Faculty of Pharmacy, Universidad Complutense de Madrid (UCM), Plaza de Ramón y Cajal, s/n, 28040 Madrid, Spain; 3 Institute of Applied Biology, Faculty of Biotechnology and Food Sciences, Slovak University of Agriculture in Nitra, Trieda Andreja Hlinku 2, 949 76 Nitra, Slovakia; xdupak@uniag.sk

**Keywords:** inflammation, oxidative stress, 3T3-L1, coffee metabolites, dihydrocaffeic acid, dihydroferulic acid, hydroxyhippuric acid, obesity, adipocytes, IL-6, IL-1β, MCP-1

## Abstract

Obesity is coupled with an altered redox state and low-level inflammation. Oxidative stress may increase pre-adipocyte proliferation, adipocyte differentiation and mature adipocyte size. Regarding inflammation, the dysregulation of cytokine production by adipose tissue takes place in obesity, which is promoted by oxidative stress. Polyphenols may exert a positive effect on obesity, not only by modulating the redox state, but also due to their anti-inflammatory activity. Coffee, which is one of the most consumed beverages, is very rich in phenolic compounds. Bioavailability studies on coffee phenols have shown that the most abundant group of metabolites in plasma and urine are dihydrocaffeic (DHCA), dihydroferulic (DHFA), and hydroxyhippuric (HHA) acids, the three acids of colonic origin. To better understand the antioxidant and anti-inflammatory properties of DHCA, DHFA, and HHA, an inflammation/oxidation model was set up in the pre-adipocyte 3T3-L1 cell line using tumor necrosis factor-α (TNF-α). After the exposure of 3T3-L1 cells to 0.5, 1, 5, and 10 µM of TNF-α at different times, the cell viability, interleukin (IL)-6 secretion, and the production of reactive oxygen species (ROS) and glutathione (GSH) were determined. Using the TNF-α prooxidant and proinflammatory conditions established (10 µM, 24 h), it was observed that the physiological concentrations (0.5, 1, 5, and 10 µM) of DHCA, DHFA, and HHA induced dose-dependent antioxidant effects according to the ROS, GSH, and antioxidant enzyme (glutathione peroxidase) results. In addition, reductions in the IL-1β, IL-6, and monocyte chemoattractant protein-1 (MCP-1) concentrations were observed to different extents depending on the metabolite (DHFA, HHA, or DHCA) and the concentration used. In conclusion, the main colonic metabolites from coffee chlorogenic acids may counteract TNF-α-induced inflammation and oxidative stress in the 3T3-L1 cell line, and thus, they present antiobesity potential.

## 1. Introduction

Obesity has become a major public health problem worldwide, affecting persons of all ages and socio-economic backgrounds. Obesity is characterized by a state of chronic, low-grade inflammation, which may act as the potential link between adipose tissue expansion and obesity-induced health complications, as the adipose tissue is an active endocrine organ that is capable of producing cytokines and adipokines like tumor necrosis factor-α (TNF-α), interleukin (IL) 6, leptin, resistin, and adiponectin [1,2]. Additionally, oxidative stress may also be a mechanistic link between obesity and obesity-related complications. People with obesity not only show enhanced levels of reactive oxygen species (ROS) or nitrogen species but also lower antioxidant defenses than their normal-weight counterparts, with antioxidant defenses inversely correlated with central adiposity [3]. A recent study by Sies et al., 2022 supports that oxidative stress, resulting from exaggerated ROS production that is unmanageable by cellular antioxidant defenses, together with the inflammatory and immune responses, plays a critical role in cell pathogenesis [4]. Although ROS act as signaling messengers that activate inflammatory pathways through protein kinases, transcription factors, and the increased genomic expressions of proinflammatory factors [4,5], sustained ROS over-generation is involved in the onset of severe chronic diseases with an inflammatory component. Thus, during the inflammatory process, the production of pro-inflammatory cytokines is usually accompanied by oxidative stress [6,7], confirming the intimate pathophysiological relation between inflammation and oxidative stress [8,9]. The activation of both processes provokes changes in the structure and function of cellular lipids and proteins, mechanisms of cell transport, Toll-like receptor (TLR), and immune system cells that lead to the amplified production of pro-inflammatory cytokines and cell injury [10].

To prevent the over-generation of ROS and, consequently, oxidative stress and its associated diseases, the cell displays an antioxidant defense system, which comprises non-enzymatic (classically reduced glutathione (GSH)) and enzymatic (glutathione peroxidase (GPx), glutathione reductase (GR), glutathione-S-transferase (GST), and catalase (CAT), among others) components [4,11]. Nevertheless, exogenous natural antioxidants, such as plant polyphenols, may reinforce the endogenous antioxidant defense system and restore optimal balance by neutralizing ROS and improving defenses [12,13,14]. Apropos of the referred joint responses between oxidative stress and inflammation, the search for dietary compounds with antioxidant and anti-inflammatory activities as a natural alternative for the prevention of inflammation-related diseases has received significant attention during the past decade [15]. Coffee is a major source of phenolic compounds in Western countries [16], and its moderate consumption may have anti-inflammatory effects [17]. Cell culture studies suggest that the modest but significant anti-inflammatory effects of coffee could be mediated by hydroxycinnamic (chlorogenic) acids, the major phenolic component that has been proven to suppress the expressions of interleukins and cell adhesion molecules (vascular (VCAM-1) and intercellular (ICAM-1) cell adhesion molecules and E-selectin) induced by IL-1β [18] or lipopolysaccharide (LPS) [19].

Due to the low bioavailability of main coffee polyphenols, particularly hydroxycinnamic acids, most recent research is focusing on their microbial-derived metabolites as the main contributors for the antioxidant and anti-inflammatory effects of their phenolic precursors [20,21,22,23]. A previous bioavailability study in humans carried out by our group showed that the main circulating metabolites after the consumption of a green/roasted coffee blend were phase II conjugated forms of dihydrocaffeic, dihydroferulic, and dihydrocoumaric acids resulting from the microbial reduction of caffeic, ferulic, and p-coumaric acids produced after the hydrolysis of coffee hydroxycinnamic acids by esterases in the small intestine [24]. These results were confirmed some years later in another human study using a decaffeinated green coffee phenolic extract (GCPE) nutraceutical [25]. Both bioavailability studies exposed that the most abundant groups of coffee phenol catabolites were feruloylglycine, along with dihydrohydroxycinnamic acids and their phase II derivatives, mainly dihydroferulic acid (DHFA) and dihydrocaffeic acid (DHCA), reaching concentrations in plasma within the micromolar range 5–10 h after the intake of the hydroxycinnamic source [24,25,26,27]. Additionally, up to 10 µM of hydroxyhippuric acid (HHA) was present in both urine and plasma after 24 h of coffee intake [24]. Interestingly, after the regular intake of the GCPE nutraceutical for 8 weeks, the concentration of HHA in urine raised from 32 µmoles at the baseline to 46 µmoles at week 8, becoming the most abundant metabolite quantified in urine [25]. These precedents sustain the selection of the three colonic-derived coffee metabolites used in the present study, whose structures are shown in Supplementary Figure S1.

Microbial metabolites can be absorbed in the colon and reach all tissues and organs through systemic circulation; thus, it is necessary to study their potential bioactivity in all cell types that may be targeted. We previously demonstrated that microbial metabolites from hydroxycinnamic acids protect human EA.hy926 endothelial cells from TNF-α induced inflammation [28], and recently, the same effect was confirmed in hepatic HepG2 cells [23]. Consequently, it is interesting to study the antioxidant/anti-inflammatory effects of these abundant coffee colonic metabolites in 3T3-L1 cells to understand if they could play a role in combating obesity. Therefore, the objective of the present study was to test the potential antioxidant/anti-inflammatory effects of the three main colonic metabolites from coffee hydroxycinnamic acids, DHFA, DHCA, and HHA, on cells from connective tissue. To this end, firstly, the model of inflammation/oxidative stress induced by TNF-α recently developed in hepatic and endothelial cells was established in an embryonic fibroblast cell line, 3T3-L1, originating from a primary cell culture with a long-life span and which can be passaged indefinitely, in contrast to the primary cell culture, in which cells are directly isolated from the parental tissue of interest. These cells are commonly used for analyzing subcellular pathways involved in preadipocyte cell differentiation [29,30]. For instance, recently, some authors [31] used the 3T3-L1 preadipocyte model to assess the bioactivity of 6-shogaol and showed that this compound significantly inhibited proliferation and differentiation. However, this is the first study, to our knowledge, that has addressed the potential chemo-prevention of DHFA, DHCA, and HHA against inflammation and oxidation in the 3T3-L1 cell line model, and to that end, we evaluated the production of inflammatory mediators such as IL-1β, IL-6, and monocyte chemoattractant protein-1 (MCP-1), together with redox markers such as ROS, GSH, GPx, and GR.

## 2. Results

### 2.1. Establishment of the Inflammation and Oxidation Model in 3T3-L1 Fibroblasts

**Figure 1:**
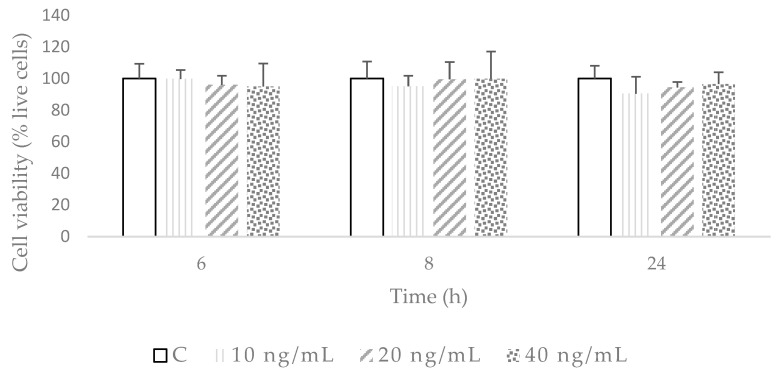
Cell viability after exposure to 10, 20, and 40 ng/mL of TNF-α during 6, 8, and 24 h in 3T3-L1 cell line. Results, expressed as percentage of live cells, are shown as means ± SD (*n* = 4–6). According to ANOVA, there were no statistically significant differences within the different times and the different concentrations (*p* < 0.05).

**Figure 2:**
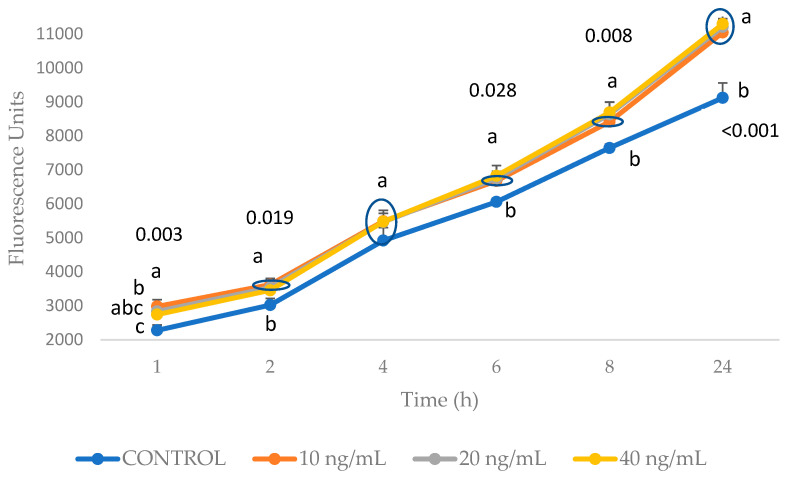
Production of reactive oxygen species (ROS) after exposure to 10, 20, and 40 ng/mL of TNF-α during 1, 2, 4, 6, 8, and 24 h in 3T3-L1 cell line cultures. Results, expressed as fluorescence units, are shown as means ± SD (*n* = 4–6). P values indicate differences within the same hour according to ANOVA. Different letters indicate statistical differences within the same hour according to the Bonferroni test (*p* < 0.05).

To study the antioxidant and anti-inflammatory effects of the DHCA, DHFA, and HHA coffee metabolites in the 3T3-L1 fibroblast cell line, a cell culture model of TNF-α-stimulated cells, similar to that previously used in hepatoma HepG2 cells [23] and endothelial EA.hy926 cells [28], was adapted to cultured 3T3-L1 cells. Firstly, the effects on the cell viability of TNF-α at the concentrations selected (10, 20, and 40 ng/mL) at 6, 8, and 24 h were tested (Figure 1). The results show that these conditions did not induce cell death. Then, the 3T3-L1 cells were exposed to TNF-α at 10, 20, and 40 ng/mL, and the production of ROS was measured from 1 to 24 h (Figure 2). The time course ROS production experiment revealed that dichlorofluorescein (DCFH) fluorescence gradually increased to 24 h with the three TNF-α treatments as follows: after 1 h, the ROS production after treatment with concentrations of 10 (*p* = 0.002) and 20 ng/mL (*p* = 0.019) of TNF-α were statistically higher than the control; in contrast, after 2 h, only the 10 ng/mL treatment had a higher result (*p* = 0.017) than the control, and after 4 h, there were no statistical differences among the treatments and control. After 6 h, the ROS levels were higher (*p* = 0.027) only with the 40 ng/mL TNF-α concentration, and after 8 h with 20 (*p* = 0.013) and 40 ng/mL (*p* = 0.009), and after 24 h, the three concentrations showed significantly higher (*p* = 0.001) ROS production levels than the control, with no differences among the three TNF-α concentrations tested. Bearing in mind these results, from the oxidation point of view, the condition of TNF-α at 10 ng/mL for 24 h was selected since it was the lowest concentration of TNF-α, resembling a more physiological condition. Afterwards, to understand the effects on inflammation of 10 ng/mL of TNF-α for 24 h in 3T3-L1 pre-adipocytes, the cells were exposed to this condition, and the proinflammatory molecule, IL-6, was measured. We observed that the levels of IL-6 significantly increased (*p* = 0.017) after the TNF-α treatment (control cells (mean ± standard deviation): 47.49 ± 9.99 pg/mL; after 10 ng/mL of TNF-α treatment: 125.01 ± 2.50 pg/mL), showing the ability of this TNF-α treatment under these conditions to induce inflammation in 3T3-L1 cells.

### 2.2. Direct Effects of DHCA, DHFA, and HHA on 3T3-L1 Cell Line Viability and ROS Production

**Figure 3:**
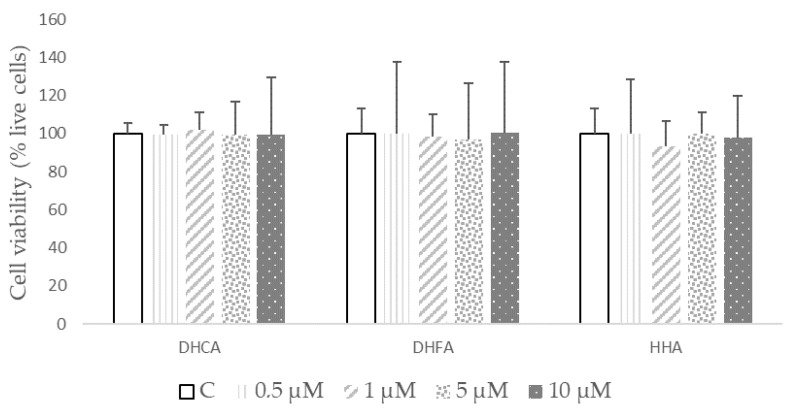
Cell viability after treatment of dihydrocaffeic acid (DHCA), dihydroferulic acid (DHFA), or hydroxyhippuric acid (HHA) at 0 (C), 0.5, 1, 5, and 10 μM for 24 h in 3T3-L1 cell line. Results, expressed as percentage of live cells, are shown as means ± SD (*n* = 4). According to ANOVA, there were no statistically significant differences within the different concentrations of each metabolite (*p* < 0.05).

According to the crystal violet results depicted in Figure 3, none of the studied doses of the coffee metabolites DHCA, DHFA, and HHA evoked cytotoxic effects after 24 h, ensuring that the range of concentrations selected to evaluate the antioxidant and anti-inflammatory effects was safe during the period of treatment.

In the same line, it was essential to guarantee that the tested doses of coffee metabolites after 24 h did not substantially modify per se the steady-state redox status of the 3T3-L1 cells. Table 1 shows that none of the concentrations of any compound altered the basal production of ROS, confirming the basal redox condition of 3T3-L1 fibroblasts.

### 2.3. Protective Effects of DHCA, DHFA, and HHA on 3T3-L1 Cell Line on IL-1β, IL-6, and MCP-1 Inflammation Markers

**Figure 4:**
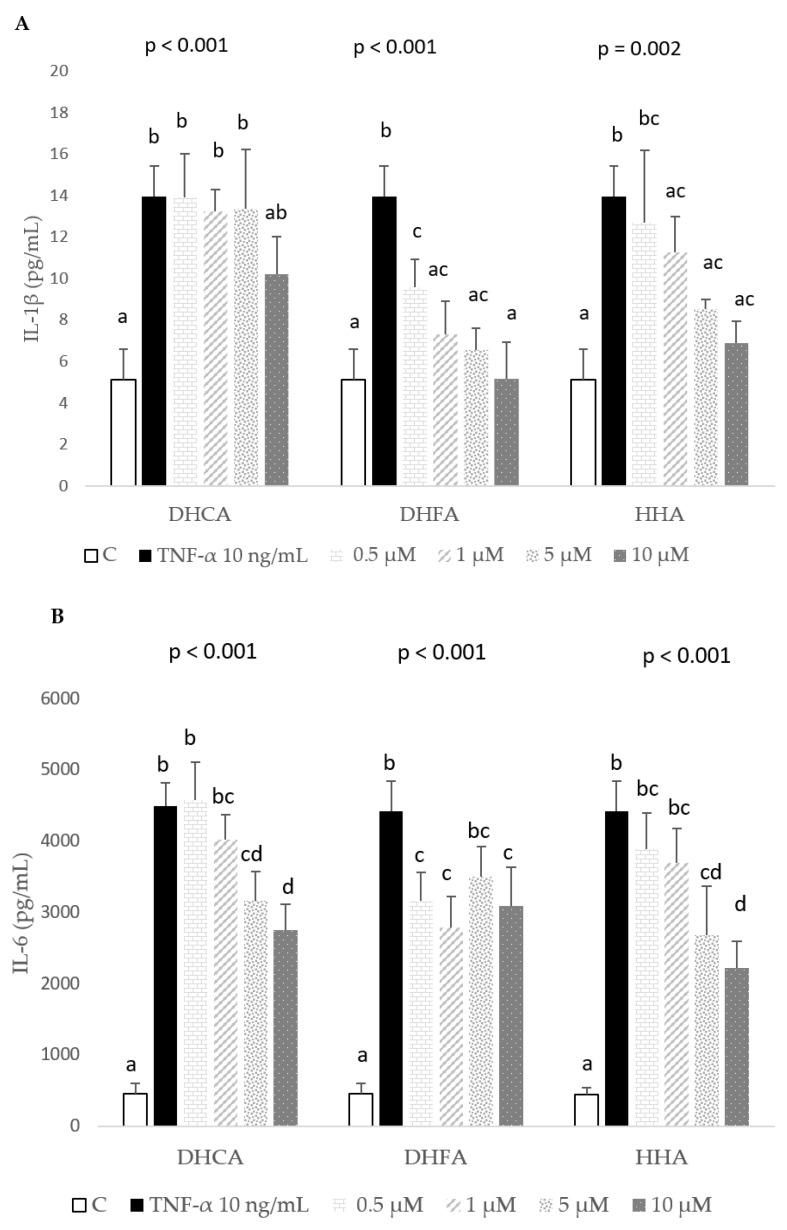

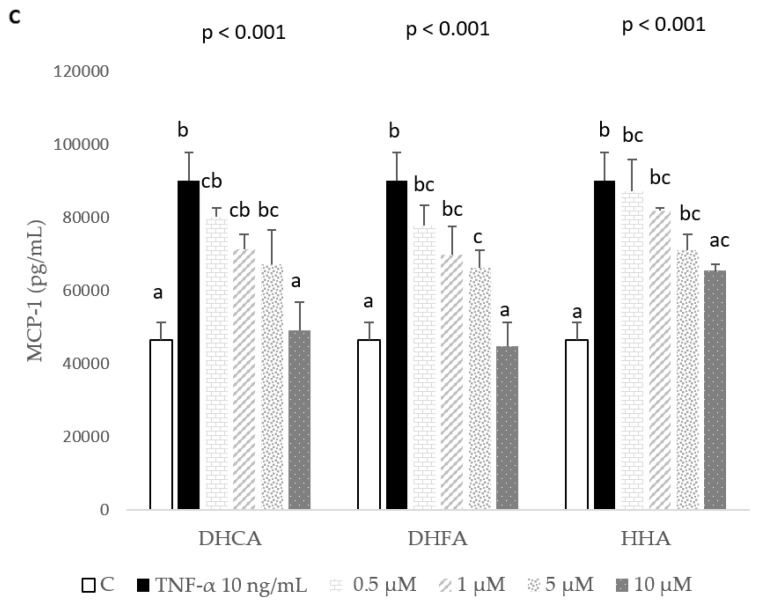
Effect of co-treatment of 0 (C), 0.5, 1, 5, and 10 μM dihydrocaffeic acid (DHCA), dihydroferulic acid (DHFA), and hydroxyhippuric acid (HHA) with 10 ng/mL TNF-α over 24 h on the production of (**A**) interleukin-1β (IL-1β), (**B**) interleukin-6 (IL-6), and (**C**) monocyte chemoattractant protein-1 (MCP-1) in 3T3-L1 cell line. Results represent means ± SD (*n* = 3–4), with *p*-values showing the results from ANOVA. Different letters indicate statistical differences according to paired Bonferroni test within the same compound (*p* < 0.05).

The potential anti-inflammatory capacity of the three coffee metabolites was tested through the determination of inflammatory mediators IL-1β, IL-6, and MCP-1 (Figure 4A, Figure 4B, and Figure 4C, respectively). Regarding the effects on IL-1β, DHCA did not reduce the concentration of this cytokine, in contrast to DHFA and HHA, which decreased the levels in a dose-dependent manner. Particularly, DHFA showed high activity, since even at the lowest concentration tested (0.5 µM), statistically lower levels of IL-1β compared to that induced by TNF-α (10 ng/mL, 24 h) were observed. Regarding IL-6, DHCA and HHA diminished this cytokine, although only the higher concentrations tested (5 and 10 µM) were different from the negative control (TNF-α). Contrarily, DHFA showed clear anti-inflammatory effects at 0.5, 1, and 10 µM. Finally, the three metabolites at 10 µM returned MCP-1 levels to close to that of untreated controls.

### 2.4. Protective Effects of DHCA, DHFA, and HHA on Oxidative Stress Markers in 3T3-L1 Cells

The co-treatment of 3T3-L1 cells with different concentrations (0.5, 1, 5, and 10 µM) of the assessed metabolites was carried out to determine their effects on the established oxidative stress cell model, measuring the response of the antioxidant enzymes (GPx and GR) as well as the ROS and GSH levels.

**Figure 5:**
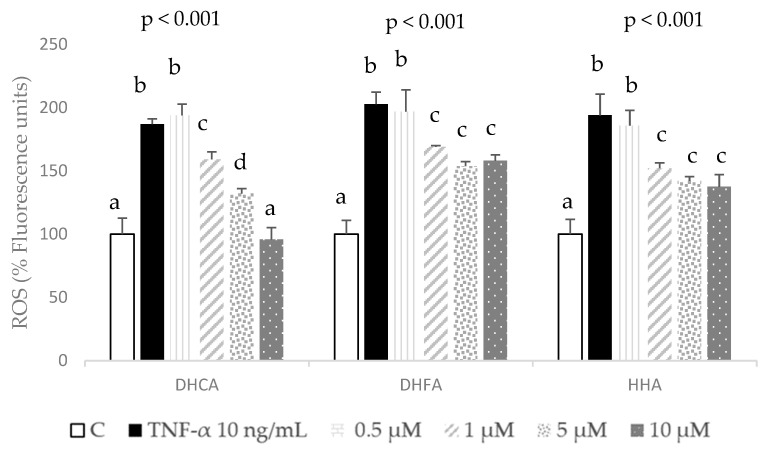
Effect of co-treatment of 0 (C), 0.5, 1, 5, and 10 μM dihydrocaffeic acid (DHCA), dihydroferulic acid (DHFA), and hydroxyhippuric acid (HHA) with 10 ng/mL TNF-α over 24 h on the production of reactive oxygen species (ROS). Data represent means ± SD (*n* = 4) per condition. ANOVA results are shown above the figures (*p* values), and different letters indicate statistical differences according to paired Bonferroni test within the same compound (*p* < 0.05).

A remarkable dose-dependent decrease in ROS production was observed with DHCA, which showed a complete recovery of TNF-α-induced ROS to the basal unstressed cells’ levels with the highest dose tested, 10 µM (Figure 5). Both DHFA and HHA also showed a significant reduction of ROS production induced by TNF-α, but this recovery was partial and not dose-dependent, since it was similar for the three highest concentrations, 1, 5, and 10 µM. No significant change in ROS generation was found with 0.5 µM of any of the tested compounds (Figure 5). The results indicate that co-treatment with colonic metabolites from coffee significantly prevents or decreases the enhanced generation of ROS induced by TNF-α. Considering the lack of effect on the ROS levels of the lowest concentration (0.5 µM) of assessed metabolites, this dose was not further assayed in the GSH and antioxidant enzyme measurements.

**Figure 6:**
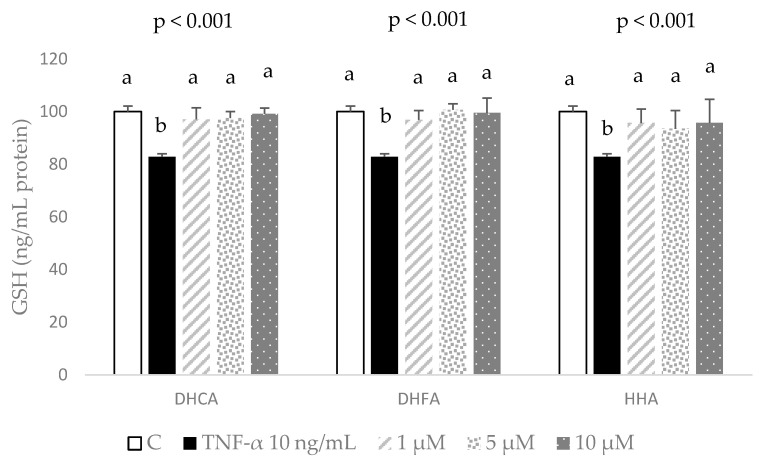
Effect of co-treatment of 0 (C), 1, 5, and 10 μM dihydrocaffeic acid (DHCA), dihydroferulic acid (DHFA), and hydroxyhippuric acid (HHA) with 10 ng/mL TNF-α over 24 h on the production of reduced glutathione (GSH). Data are shown as means ± SD (*n*= 3–4) per condition. ANOVA results are shown above the figures (*p* values), and different letters indicate statistical differences according to paired Bonferroni test within the same compound (*p* < 0.05).

The main biomarker of the non-enzymatic antioxidant defenses, GSH, was determined in the 3T3-L1 cell line submitted to 10 ng/mL of TNF-α for 24 h. This treatment evoked a 20% decrease in the cellular concentration of GSH, indicative of an increased utilization of the major cell reducing power. As depicted in Figure 6, this hazardous TNF-α-induced decline in GSH concentration in the 3T3-L1 cell line was completely recovered by a co-treatment of all three concentrations of the three metabolites. Remarkably, even the lowest concentration of 1 µM from any compound was enough to implement a full return of the GSH level to that of the unstressed cells. These results indicate that even low concentrations of all three coffee metabolites prevent the cell challenging depletion of GSH induced by TNF-α.

**Figure 7:**
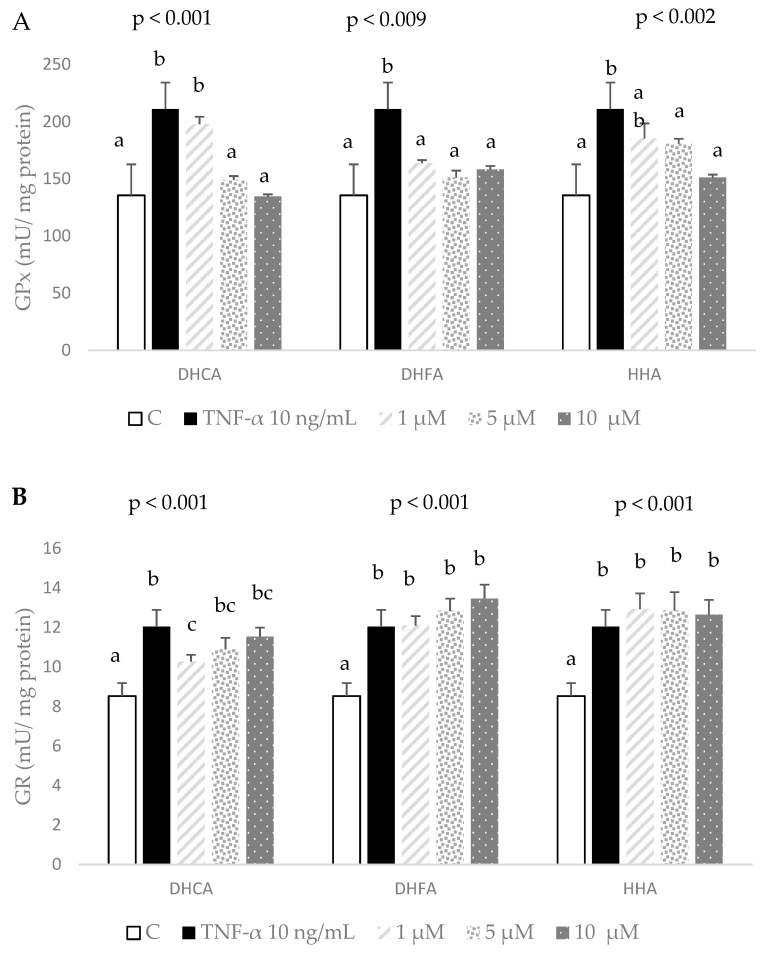
Effect of co-treatment of 0 (C), 1, 5, and 10 μM dihydrocaffeic acid (DHCA), dihydroferulic acid (DHFA), and hydroxyhippuric acid (HHA) with 10 ng/mL TNF-α over 24 h on the activities of glutathione peroxidase (GPx (**A**)) and glutathione reductase (GR (**B**)). Results represent means ± SD (*n* = 3–4), with *p*-values showing the results from ANOVA. Different letters indicate statistical differences according to paired Bonferroni test within the same compound (*p* < 0.05).

Finally, the activities of enzymatic antioxidant defenses, GPx and GR, were evaluated as representative responses of the antioxidant enzyme system to a stressful insult. When 3T3-L1 fibroblasts were submitted to 10 ng/mL of TNF-α for 24 h, a significant enhancement of around 40–50% in GPx and GR activities was observed, indicating a positive response of the antioxidant system to quench over-generated ROS (Figure 5). Remarkably, co-treatment with all three metabolites dose-dependently reduced the TNF-α-induced increase in GPx, reducing the waste of the antioxidant system (Figure 7A). It is worth noting that 5–10 µM of all three compounds completely returned the GPx activity level to that of the control 3T3-L1 cells at the end of the stress period.

Contrary to what was found in GPx, only one condition, 1 µM DHCA, evoked a partial but significant decrease in the enhanced GR activity. Surprisingly, none of the other tested concentrations of the three colonic metabolites was able to significantly reduce the TNF-α-induced increased GR activity (Figure 7B).

## 3. Discussion

The most recent research on the chemo-protective effect of natural antioxidants has focused on the contribution of colonic metabolites from major dietary polyphenols [20,21,22]. Since the bioavailability of dietary phenolic compounds is usually limited, a great proportion of ingested polyphenols arrives unaltered to the colon where the microbiota generates a qualitative and quantitative variety of metabolites with bioactive potential [20,21,22]. Coffee is among the richest sources of dietary polyphenols, particularly hydroxycinnamic (chlorogenic) acids, which are important precursors of bioactive metabolites. In fact, our research team [23,28,32] and other groups have previously reported the bioactive potential of metabolites from hydroxycinnamic acids. Thus, the next step was to study whether this protective effect was applicable to other cell types, such as the mesenchymal lineage, in order to claim a systemic protection of cell viability and function by these colonic-derived compounds. In this study, we prove the cyto-protective effects of three main colonic metabolites from coffee, DHCA, DHFA, and HHA, in 3T3-L1 fibroblasts exposed to a condition of oxidative stress and inflammation induced by TNF-α.

Since oxidative stress and inflammation are intimately related to the onset and development of many degenerative diseases involving different tissues, it is crucial to study the potential antioxidative and anti-inflammatory effects of specific dietary compounds and their main metabolites in as many as cell types and tissues as possible. Thus, major coffee phenolic compounds and hydroxycinnamic acids have been proven to be effective in the nutritional prevention of cardiovascular diseases, metabolic syndrome, type II diabetes mellitus, and different types of cancer [33]. As stated above, the contribution to the health effects of the ingested compounds and that of their colonic metabolites is currently the subject of study, and the primary step is to investigate the specific effects of particular compounds and metabolites on cell viability and function in cell types and lines from different origins submitted to harmful conditions. In particular, this is the first time that the potential protective effects of the three major colonic metabolites from coffee chlorogenic acids, DHCA, DHFA, and HHA, have been assayed in cultured fibroblasts. This study is essential because fibrosis, which may occur in all organs and is particularly clinically relevant in major solid organs, involves fibroblast activation by oxidative stress and/or inflammation [34]. Indeed, studies have reported that UVA exposure significantly leads to ROS accumulation in keratinocytes, melanocytes, and fibroblasts that are responsible for oxidative damage to biomolecules, including DNA, lipids, and proteins in the skin, which are associated with the photo-aging process [35].

**Figure 8:**
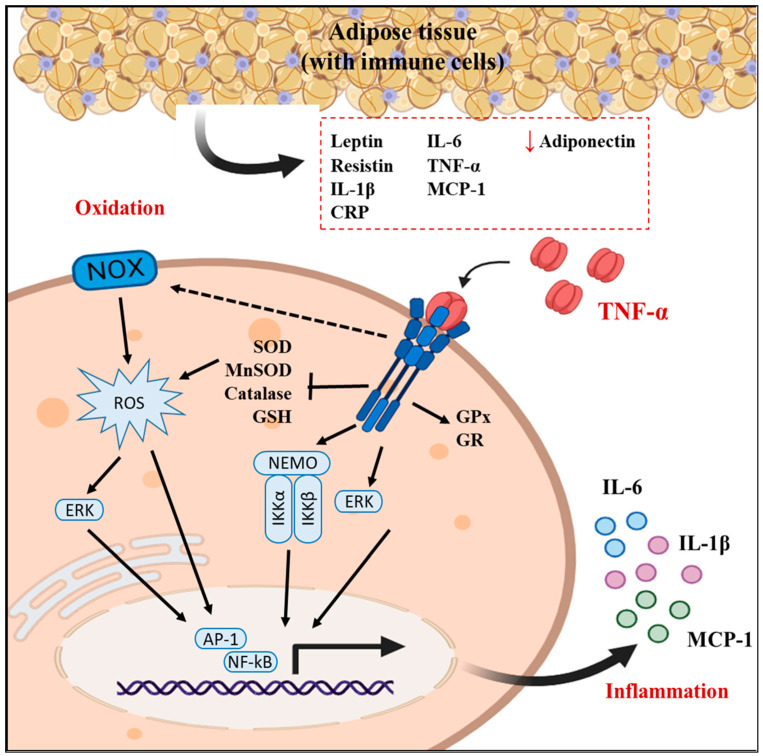
Effects of Tumour Necrosis Factor-α (TNF-α) on 3T3-L1 cellular targets involved in the pro-inflammatory and pro-oxidant effects stated in the present study.

TNF-α has a key role in the inflammatory process among other obesity-related metabolic disturbances [36]. In the 3T3-L1 cell line, TNF-α was shown to dose-dependently suppress the activities of CuZnSOD and MnSOD, which, in turn, may lead to an increase in the production of active free radicals, and that of catalase, which may imply peroxisomal dysfunction. In addition, the intracellular concentrations of GSH and protein thiol are decreased by TNF-α, suggesting that TNF-α enhances oxidative stress ([36]; Figure 8). Furthermore, a high concentration of TNF-α may alter lipid homeostasis in adipocytes [37] as well as provoke oxidative stress in hepatic cells [23] and inflammation in endothelial cells [28,38]. Previous studies were considered to establish reliable conditions for a model of TNF-α-induced inflammation and oxidative stress in 3T3-L1 fibroblasts; thus, TNF-α has been used in different cell lines at different conditions ranging from 6 ng/mL for 30 min [32], to 10 ng/mL at 2 to 24 h [28,39,40], to 40 ng/mL for 6 h [23], or even 50 ng/mL for 18 h, which induced a significant increase in IL-6 in endothelial EA.hy926 cells [38]. Based on the results obtained for the redox and inflammation biomarkers, in the present study, the condition selected for the 3T3-L1 fibroblasts was 10 ng/mL, which is somewhat lower than the value that was recently proven to be optimum in hepatic cells [23], which was 40 ng/mL, although a longer exposure is required to ensure significantly high ROS production (see Figure 2). It is necessary to remark that a direct anti-inflammatory/antioxidant action may be expected in our assay, since 3T3-L1 cells are submitted to the combined treatment of the coffee metabolites and TNF-α.

Chronic inflammation is an important component of many diseases, including obesity, atherosclerosis, diabetes, metabolic disorders, cancer, and autoimmune conditions. In these diseases, increased proinflammatory factors, such as IL-1β, which is secreted by several different cell types and is one of the key cytokines involved in diabetic inflammation; IL-6, expressed and secreted by macrophages and adipocytes, which are observed in obesity-related diseases; and MCP-1, also secreted by macrophages and adipocytes, which is important in regulating inflammation, play important roles [2]. TNF-α induces the expressions of several cytokines in the 3T3-L1 cell line, which may lead to the up-regulation of other inflammatory mediators [2,41]. In agreement, in the present work, the exposure of 3T3-L1 fibroblasts to TNF-α at 10 ng/mL for 24 h enhanced the production of IL-1β, IL-6, and MCP-1, indicating an active inflammatory process. The colonic metabolites studied showed anti-inflammatory effects to different extents depending on their chemical structures and concentrations. DHCA did not reduce the concentration of IL-1β; however, at the highest concentration tested (10 µM), it lowered the concentrations of IL-6 and MCP-1. On the contrary, DHFA showed clear anti-inflammatory effects, as it decreased the levels of IL-1 β and IL-6 at all of the tested concentrations, from 0.5 to 10 µM. These results partially agree with those obtained by Sánchez-Medina et al. [23] in HepG2 cells, as DHFA was the metabolite that showed more effective anti-inflammatory properties, reducing the levels of IL-6, macrophage inflammatory protein-1β (MIP-1β), and MCP-1 at 5 and 10 µM. However, on the other hand, DHCA in the HepG2 cells did not induce changes in the production of IL-6, MIP-1β, MCP-1, and IL-8 [23], in contrast to the anti-inflammatory effects observed here on MCP-1 and IL-6 at 10 µM. Other polyphenols were revealed to reduce inflammation, such as resveratrol that attenuated IL-6, plasminogen activator inhibitor-1 (PAI-1), and adiponectin levels in 3T3-L1 adipocytes when added with TNF-α [42], as well as MCP-1 production and gene expression in 3T3-L1 treated with TNF-α [43].

It is worth highlighting the effect of HHA as a biologically active metabolite, which has been much less studied than DHCA and DHFA. To the best of our knowledge, this is the first study on the bioactivity of this colonic metabolite in the 3T3-L1 cell line, and clear anti-inflammatory effects on IL-1β, IL-6, and MCP-1 at 10 µM were observed (Figure 4A–C). These results support the anti-inflammatory properties of HHA described in two previous reports, one showing the effect of HHA tested at 1 μM (within the range of 0.1–10 μM), which reduced the secretions of TNF-α, IL-1β, and IL-6 in LPS-stimulated peripheral blood mononuclear cells from healthy subjects [44], and the other study showing that HHA decreased IL-8 at 0.5 up to 10 µM and MIP-1β at 10 µM in HepG2 cells [23]. As pointed out in the study by Sanchez-Medina et al. [23], the fact that high levels of HHA are observed in plasma and urine after the intake of hydroxycinnamic acids from coffee, flavonoids such as flavan-3-ols from cocoa and nuts, citrus flavanones, or anthocyanins in berries, among other fruits, support the biological relevance of the present results.

As aforementioned, oxidative stress may also be a mechanistic link between obesity and obesity-related complications. In hepatic-like cells submitted to tertbutyl hydroperoxide (*t*-BOOH), pre-treatment with 0.2–10 µM of DHCA markedly prevented cytotoxicity, recovered diminished levels of GSH, as well as normalized the ROS levels and antioxidant enzyme activity [32], whereas only a slight protection against cell cytotoxicity, lipid oxidation, and GSH depletion was observed with DHFA [32]. More recently, in an experimental model of oxidative stress/inflammation induced by TNF-α, as in the present study, all three microbial compounds, DHCA, DHFA, and HHA, showed significant chemo-protective effects in hepatic HepG2 viability and function [23].

A balanced ROS concentration is crucial for cell function, and increased ROS generation is a reliable index of the oxidative damage caused to living cells. Consequently, increased ROS production in 3T3-L1 cells upon treatment with TNF-α denotes a condition of acute oxidative stress that, if sustained, might evoke irreversible cell damage. In the present study, the co-treatment of a TNF-α-stressed 3T3-L1 cell line with coffee metabolites significantly decreased the over-production of ROS, confirming, in fibroblasts, the ROS quenching capacity of the compounds already reported in hepatic cells [23]. Phenolic molecules are characterized by the presence of an aromatic ring with one or more hydroxyl groups that provides reducing power [15,23]; thus, the results suggest that, acting as powerful antioxidants, coffee metabolites prevent or reduce cell oxidative damage. These findings confirm previous data reporting a protective effect of colonic coffee metabolites from phenolic compounds in endothelial cells [28], as well as cocoa colonic metabolites in endothelial, pancreatic beta, renal proximal tubular, and cardiac cell lines [15]. Thus, we may conclude that the coffee colonic metabolites DHCA, DHFA, and HHA could restrain the stress-induced production of ROS as an early response of the antioxidant response against oxidative stress.

GSH is instrumental to cope with a pro-oxidant condition both as directly reducing power donating protons to reactive oxygen and nitrogen radicals and as a co-enzyme of GPx and glutathione-S-transferase; hence, decreased levels of GSH indicate increased intracellular oxidation [45]. Thus, the declined GSH concentration induced by TNF-α indicates a situation of imminent oxidative stress that will provoke irreversible oxidative damage to proteins, lipids, and nucleic acids. This harmful state was entirely prevented through co-treatment with coffee metabolites for 24 h, similarly to the chemo-protective effects previously reported for hydroxycinnamic acid colonic metabolites in hepatic [32] as well as endothelial cells [28]. This outcome is crucial because, while facing a stressful challenge, preserving the GSH concentration over a critical value represents a vital advantage for cell survival.

In addition to GSH, the other branch of the antioxidant defense system is composed of antioxidant enzymes such as GPx and GR. GPx catalyzes the reduction in cell-harming peroxide species using GSH as a co-enzyme that is converted to oxidized glutathione; then, GR effectively recovers oxidized glutathione back to GSH, enabling a balanced concentration of cellular GSH [45]. After 24 h of treatment with 10 µM of TNF-α, the substantial rises in the activities of GPx and GR observed in the 3T3-L1 fibroblasts unambiguously indicate a positive response of the cell defense system to face an over-production of ROS induced by the oxidative insult. However, an unremitting activity of antioxidant enzymes would suffocate the defense system and hamper a dependable response to future stress challenges. Although we have no plausible explanation for the lack of recovery of GR activity to basal levels with any metabolite treatment, we speculate that the GR activity is usually lower than GPx, and management by GR of the GPx-induced over-generation takes longer to process. Nonetheless, the GPx activity (first antioxidant barrier to reactive hydroperoxides) of cells co-treated with chlorogenic acid metabolites safely returned to a steady-state condition at the end of an induced challenge, lessening cell damage and thus permitting the cell to face further oxidative insults in more favorable conditions. In fact, the colonic metabolite from chlorogenic acid that showed more consistent responses in the present study, DHFA, also proved to be more effective in liver HepG2 cells [23]. To sum up, the main colonic metabolites from coffee hydroxycinnamic acid may counteract TNF-α-induced inflammation and oxidative stress in the 3T3-L1 cell line, and thus present antiobesity potential. Future research could be directed towards analyzing a greater number of inflammatory biomarkers and understanding the mechanisms underlying the effects observed.

The novelty of this work is the use of the developed model of inflammation/oxidation induced by TNF-α in 3T3-L1 cells to understand the effects of DHFA, DHCA, and HHA (0.5, 1, 5, and 10 M) on cell proliferation, ROS generation, GSH, GPx, and GR, as well as IL-1β, IL-6, and MCP-1. A weakness of this work is that it is possible that interactions took place between the colonic metabolites and TNF-α in the culture media, which could have affected reactivity. Nonetheless, since the studied metabolites and TNF-α are systemically present in physiological conditions, the effects observed can be biologically relevant and may have translational application in vivo.

## 4. Materials and Methods

### 4.1. Chemicals and Reagents

From Bio Whittaker Europe (Lonza, Madrid, Spain), we acquired Dulbecco’s modified eagle’s medium (DMEM) and fetal calf serum (FCS). The three metabolites, DHCA, DHFA, and HHA; the antibiotics gentamicin, streptomycin, and penicillin; O-phthaldialdehyde (OPT); glutathione reductase (GR); reduced glutathione (GSH) and oxidized glutathione (GSSG); dichlorofluorescein diacetate (DCFH-DA); 2,4-di-nitrophenylhydrazone (DNPH); nicotinamide adenine dinucleotides phosphates (NADPH); β-mercaptoethanol; guanidine; dithiothreitol (DTT); ethylenediaminetetraacetic acid (EDTA); dimethylsulphoxide (DMSO); and sodium dodecyl sulphate (SDS) were bought from Sigma-Aldrich (Madrid-Spain). From Bio-Rad (Madrid, Spain), the Bradford reagent was purchased. TNF-α, a recombinant murine agent, was from PreproTech (Tebu-bio, Madrid, Spain). From Invitrogen by ThermoFisher Scientific (Madrid, Spain), we obtained the IL-6 ELISA kit. The cell culture dishes were obtained from Falcon (Cajal, Madrid, Spain). The 3T3-L1 cells were a donation from Dr. Angela Martinez-Valverde from the IIB Alberto Sols (CSIC-UAM). The remaining chemicals used were of analytical grade.

### 4.2. Cell Culture

Preadipocytes 3T3-L1 cells were cultured in 10% FCS supplemented DMEM F-12 medium containing 50 mg/L of gentamicin, penicillin, and streptomycin. Cells were maintained in a humidified incubator containing 5% CO_2_ and 95% air at 37 °C.

### 4.3. Induced Inflammation/Oxidation Model in 3T3-L1 Cells with TNF-α

#### 4.3.1. Pro-Inflammatory Treatment

Cells were treated with different concentrations of TNF-α (10, 20, and 40 ng/mL) for 6, 8, and 24 h in order to establish the inflammation model. These incubation times and concentrations were selected according to previous studies (see Section 3).

#### 4.3.2. Evaluation of Cell Viability

To assess cell viability, the crystal violet assay was used. Cells were seeded at low density (10^5^ cells per well) in 24-well plates, and TNF-α was added at different concentrations (10, 20, and 40 ng/mL). After treatment for 6, 8, and 24 h, cells were incubated for 20 min with crystal violet (0.2% in ethanol), and then rinsed with PBS, and 1% sodium dodecyl sulfate (SDS) was added prior to reading absorbance at 570 nm in a microplate reader (Bio-Tek, Winooski, VT, USA). The results were shown as percentage of cell viability referred to the absorbance measured in the control wells (cells treated with serum-free culture medium).

#### 4.3.3. ROS Production

DCFH assay was used to quantify ROS production. The 3T3-L1 cells were seeded in 24-well plates at 10^5^ cells/well density, incubated for 30 min with DCFH-DA, and then washed twice with PBS. TNF-α at 10, 20, and 40 ng/mL was added, and ROS production was measured from 1 to 24 h. Fluorescence emitted from the DCFH was measured in a microplate reader at an excitation wavelength of 485 nm and emission wavelength of 530 nm.

#### 4.3.4. Measurement of IL-6 Production in Cells Supernatant

Preadipocyte 3T3-L1 cells were seeded in 24-well plates (10^5^ cells per well) to assess the inflammatory effects of TNF-α in FCS medium. After 24 h incubation, TNF-α at 10 ng/mL was added for 24 h. Following that, the supernatants were collected for the measurement of IL6 using an immunoenzymatic assay, following the supplier’s protocol (Invitrogen by Thermo Fischer Scientific). Results were shown as pg/mL.

### 4.4. Direct Effects and Protective Effects against Inflammation/Oxidation of the Three Coffee Colonic Metabolites, DHCA, DHFA, and HHA, in 3T3-L1 Cells

#### 4.4.1. Direct Effects of DHCA, DHFA, and HHA in 3T3-L1 Cells

The 3T3-L1 cells were treated with different concentrations of the three coffee metabolites, DHCA, DHFA, and HHA (0.5, 1, 5, and 10 μM in 0.1% DMSO), at 6, 8, and 24 h in serum-free medium. ROS production and cell viability were assessed as described above (Section 4.3.2 and Section 4.3.3). 

#### 4.4.2. Protective Effects of DHCA, DHFA, and HHA in 3T3-L1 Cells Treated with TNF-α

To reveal the protective effects of the tested coffee metabolites against inflammation and oxidative stress, the 3T3-L1 cells were treated with TNF-α (10 ng/mL) together with DHCA, DHFA, and HHA (0.5, 1, 5, and 10 μM in 0.1% DMSO) for 24 h to measure cell viability. For ROS production, cells were treated for 2 to 24 h as described above.

#### 4.4.3. Anti-Inflammatory Effects of DHCA, DHFA, and HHA in 3T3-L1 Cells Treated with TNF-α

To assess the anti-inflammatory effects of the metabolites of coffee, pre-adipocyte 3T3-L1 cells were seeded at low density (10^5^ cells per well) in 24-well plates and incubated for 24 h with FCS + DMEM medium; then, cells were treated with DHCA, DHFA, and HHA at 0.5, 1, 5, and 10 μM and TNF-α (10 ng/mL) for 24 h. Cell supernatants were collected for the determination of IL-1β, IL-6, and MCP-1out using Bio-Plex Pro Mouse Cytokine IL-6, Bio-Plex Pro Mouse Cytokine MCP-1 (MCAF), and Bio-Plex Pro Mouse Cytokine IL-1b (Bio-Rad Laboratories Inc., Hercules, CA, USA). All analytes were measured in duplicate on a MAGPIX™ Multiplex reader fitted to a Bio-Plex ProWash Station and software Bio-Plex Manager™MP Version 1.0 (Luminex Corporation, Austin, TX, USA) for data processing. Results were expressed as pg/mL supernatant.

### 4.5. Antioxidant Effects of DHCA, DHFA, and HHA in 3T3-L1 Cells Treated with TNF-α

#### 4.5.1. Reduced Glutathione

A biomarker of oxidative stress such as the GSH content (ng/mg per protein) was used. Cells were seeded in 60 mm diameter plates (1.5 × 10^6^ cells per plate), and after incubation with the metabolites and TNF-α as described above, cells were treated with trichloroacetic acid at 5% + EDTA 2 mM for 10 min, and were later centrifuged at 3000 rpm for 30 min. Supernatants were collected for GSH determination using a fluorometric assay, measuring GSH at pH 8.0 after reaction with OPT. The excitation wavelength was at 340 nm, and the emission wavelength was at 460 nm. Finally, fluorescence data were interpolated from a standard curve of GSH (5–1000 ng), and results were expressed as ng/mg protein.

#### 4.5.2. Antioxidant Enzymes

The 3T3-L1 cells were seeded in 100 mm diameter plates (2 × 10^6^ cells/plate) and treated with the coffee metabolites and TNF-α as previously described. The determination of GPx activity was based on the oxidation of GSH by GPx using *t*-BOOH as a substrate coupled with the disappearance rate of NADPH by GR. The GR activity was evaluated by following the decrease in absorbance due to the oxidation of NADPH utilized in the reduction of oxidized glutathione. Enzyme activity was expressed as mU/mg protein. To measure the protein concentration in the sample, Bradford reagent was used.

### 4.6. Statistics

Prior to statistical analysis, the data obtained were tested for homogeneity of variances using the test of Levene. Afterwards, one-way ANOVA was applied for multiple comparisons, followed by Bonferroni test. The level of significance was set at *p* < 0.05. An SPSS version 24.0 program (SPSS Inc., Chicago, IL, USA) was used.

## 5. Conclusions

Co-treatment of DHCA, DHFA, and HHA at physiological concentrations (0.5, 1, 5, and 10 µM) with TNF-α at conditions that induce inflammation/oxidation in the 3T3-L1 cell line showed that all three colonic compounds displayed anti-inflammation and antioxidative stress effects. TNF-α induced the increase in IL-1β, IL-6, and MCP-1 levels, and these were lowered by the three metabolites to different extents depending on the compound and concentration used. ROS over-production and the higher GPx activity induced by TNF were significantly decreased, whereas GSH was preserved, when hydroxycinnamic colonic metabolites were added to the media. Our results suggest that by attenuating the TNF-α-induced changes in inflammatory cytokines and chemokine MCP-1 and by reducing oxidative stress, the studied coffee metabolites may prevent adipose tissue unbalance and thus obesity-related complications.

## Figures and Tables

**Table 1:** Effects of dihydrocaffeic acid (DHCA), dihydroferulic acid (DHFA), and hydroxyhippuric acid (HHA) at 0 (control, C), 0.5, 1, 5, and 10 μM for 24 h on the production of reactive oxygen species (ROS) in 3T3-L1 cell line.

µM	DHCA	DHFA	HHA
0 (C)	100.01 ± 2.31	100.12 ± 2.03	100.6 ± 3.6
0.5	99.7 ± 0.2	97.4 ± 3.4	97.1 ± 2.2
1	101.4 ± 1.3	97.7 ± 1.6	99.7 ± 2.2
5	101.01 ± 0.63	96.12± 1.03	96.7 ± 1.5
10	100.1 ± 1.8	95.8 ± 0.4	96.8 ± 1.7

Results, expressed as percentage of fluorescence units, are shown as means ± SD (*n* = 3). According to ANOVA results, there were no statistical differences between treatments (*p* < 0.05).

## Data Availability

The data presented in this study are available upon request from the corresponding author.

## Supplementary Materials

The following supporting information can be downloaded at: https://www.mdpi.com/article/10.3390/molecules29010088/s1, Figure S1: Chemical structure of the microbial-derived metabolites dihydrocaffeic acid (DHCA), dihydroferulic acid (DHFA) and 3-hydroxyhippuric acid (HHA).
