# Supplementary material for: Main Colonic Metabolites from Coffee Chlorogenic Acid May Counteract Tumor Necrosis Factor-α-Induced Inflammation and Oxidative Stress in 3T3-L1 Cells
Source: Molecules. 2023 Dec 22;29(1):88. doi: 10.3390/molecules29010088 (PMC10779949; doi:10.3390/molecules29010088)
Supplement: Supplementary file 1 [file molecules-29-00088-s001.zip › molecules-2718544-supplementary.pdf]

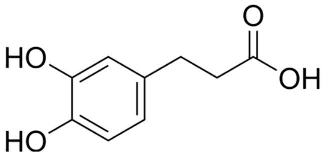

Dihydrocaffeic acid

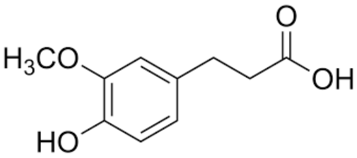

Dihydroferulic acid

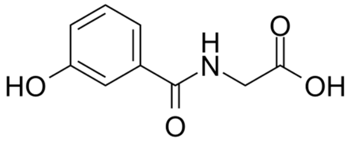

3-hydroxyhippuric acid

Supplementary Figure 1S – Chemical structure of the microbial-derived metabolites dihydrocaffeic acid (DHCA), dihydroferulic acid (DHFA) and 3-hydroxyhippuric acid (HHA)
